# Structural, Physicochemical and Digestive Property Changes of Potato Starch after Continuous and Repeated Dry Heat Modification and Its Comparative Study

**DOI:** 10.3390/foods12020335

**Published:** 2023-01-10

**Authors:** Shuangfeng Guo, Hao Wu, Xinyue Liu, Wenqing Zhao, Jiayu Zheng, Wenhao Li

**Affiliations:** College of Food Science and Engineering, Northwest A&F University, Xianyang 712100, China

**Keywords:** potato starch, dry heat treatment, physicochemical property

## Abstract

To investigate the effects of repeated dry heat treatment (RDH) and continuous dry heat treatment (CDH) on the structure and physicochemical and digestive properties of potato starch, potato starch was treated continuously and repeatedly at 130 °C for 3–18 h. The results showed that the crystalline form of starch was consistent with the original type B. Still, its physicochemical properties, such as swelling power, transparency, peak viscosity (PV), final viscosity (FV), breakdown (BD) and thermal properties (To, Tp, Tc, ΔT), tended to decrease. At the same time, solubility and RS increased after dry heat treatment. Moreover, RDH-treated starches were higher than CDH-treated ones in terms of molecular weight, crystallinity, swelling power, transparency and final viscosity for the same treatment time. Still, there was no significant difference between the thermal properties of the two. Meanwhile, the resistant starch (RS) content showed a downward trend after the peak value of 9 h of CDH treatment and five cycles of RDH treatment with increasing treatment time and the number of cycles, indicating a decrease in the overall digestibility of the starch. Overall, RDH had a more significant effect on potato starch’s structure and physicochemical properties than CDH.

## 1. Introduction

Starch is an abundant natural carbohydrate in our living diet and is present in various food sources, such as grains, tubers, and beans [1]. Starch is usually composed of two polymers: amylose with α-1,4 glycosidic linkages and amylopectin with α-1,6 linkages with a highly branched structure [2]. Potato (*Solanum tuberosum* L.) is the fourth largest food crop in the world after wheat, rice and corn [3], where starch is the main component of potatoes and has different physicochemical properties and applications than other starches. Native potato starch has larger granules, higher amylopectin content, peak viscosity, and light transmittance with good processing characteristics [4]. As a result, potato starch has several industrial uses, such as thickeners, fillers, adhesives and gelling agents [5]. However, potato starch has limitations such as being insoluble in water, unstable paste, poor transparency and solubility, and poor fluidity, which dramatically limits its application in food processing [6]. Consequently, to enhance the scope of starch applications in food production, modification of starch to improve its natural properties, including physical, chemical and enzymatic modifications, is very much possible and necessary. However, chemical modifications may leave unnecessary material behind, and enzymatic modifications are expensive [7]. Hence, the physical modification will not introduce any foreign matters into the final product while maintaining a low cost.

Dry heat treatment (DHT) is a physical modification approach where simplicity, safety and environmental friendliness are the main features [8]. Starch was treated at high temperatures in low moisture during dry heat modification for several hours. There are significant changes in the thermal properties, gelation and rheological properties of the starch [9]. It makes starch with a broader application scenario and is vital in promoting consumer upgrading. Compared with the original starch, modified starch can improve the taste and tolerance of the product to meet the processing requirements of different foods. DHT has been widely used in modifying starch raw materials such as cereals, potatoes and beans. Several studies have detected changes in the structural properties of starch after DHT. For example, Lei et al. [10] found that corn starch piled up after DHT, and speculated that this could be related to moisture loss and changes in surface properties. In addition, in a study on dry heat-treated (130 °C, 2 or 4 h) yellow millet flour and yellow millet starch, yellow millet flour and its starch also showed agglomeration in clusters [8]. Liang et al. [11] also discovered that the DHT of mung bean starch resulted in increased crystallinity of the starch compared to the native sample. DHT can destroy part of the starch granules and enhance the amylose content, thereby increasing the crystalline area of the starch granules. On the other hand, DHT can also alter the functional properties of starch, such as solubility, thermal properties and digestibility. Zou et al. [12] found that DHT treatment increased the solubility of corn starch, which was mainly related to the pores and cracks formed by starch particles under dry heat conditions, thus increasing the dissolution of amylose in amorphous regions [13]. Furthermore, when both RDS and the predicted glycemic index of high straight-chain starch were decreased when subjected to DHT at varying temperatures and times, it was obtained that DHT could be used to improve the nutritional characteristics of starch [13]. Moreover, Zou et al. [14] used waxy corn starch to prepare RDH and CDH starch at 140 °C for 20 h or 5 cycles. They concluded that the transformation temperature (T_o_, T_p_ and T_c_) and enthalpy (ΔH) value decreased, indicating that the amylose was destroyed. Additionally, the binding force of the crystal was impaired during DHT, thereby improving the starch gelatinization properties.

DHT includes continuous dry heat (CDH) treatment and repeated dry heat (RDH) treatment. Most researchers have mainly focused on the study of CDH of starch. Nevertheless, the interaction between starch molecules easily reaches the equilibrium point in the CDH treatment, limiting to a certain extent the modification of starch properties [15]. However, RDH may be more effective in altering the properties of starch. A study investigated the impact of DHT on sweet potato starch and found that RDH treatment markedly changed the structural, physicochemical and digestive properties of sweet potato starch compared to CDH treatment [15]. Furthermore, Zhou et al. [16] also studied quinoa starch to CDH and RDH treatments, respectively. The water solubility, swelling power, paste viscosity and thermal parameters (T_o_, T_p_, T_c_, ΔT) of RDH starch samples were less compared to CDH starch. Zhang et al. [17] found that, compared to the native starch, the resistant starch (RS) content of wheat starch increased, while the SDS content tended to increase and then decrease with the number of cycles and/or treatment time. Overall, RDH treatment had better results in changing the physicochemical properties of quinoa starch. Although CDH and RDH treatments of some starches have been studied, potato starch has not been studied in depth, especially since the mechanical changes of potato starch during RDH treatment are not fully understood. Therefore, in this work, potato starch was considered an object of study to investigate the changes in the structure and physicochemical properties of starch treated with RDH and CDH, and to evaluate and compare the impact of RDH and CDH treatment on its properties under the same treatment time.

## 2. Materials and Methods

### 2.1. Materials

Potato starch was obtained from the Aladdin reagent company. It contains 27.38% amylose, 0.13% crude protein, 0.38% fat and 0.23% ash. 8-Hydroxypyrene-1,3,6-trisulfonic acid trisodium salt (ABTS), Glycosylase (2500 U/mL) and Porcine Pancreatic Alpha-Amylase (USP grade) were provided by Aladdin Bio-chem Technology Co., LTD., Shanghai, China. All other reagents were of analytical grade, and purchased from Sanli Chemical Reagent, Yangling, China.

### 2.2. Dry-Heat Treatments

The starch was dried in a 45 °C oven for 13 h until the starch moisture content was lower than 10% and then passed through a 100 mesh sieve. For RDH treatment, the starch was placed in the oven at 130 °C for 3 h, cooled naturally to room temperature 25 °C, making the cooling rate 3.5 °C/min and passed the 100 mesh sieve again; that was, the starch with RDH cycle was obtained (RDH-1). The starch RDH-2, RDH-3, RDH-4, RDH-5 and RDH-6 were labeled after repeated treatment 2 to 6 times. For the CDH treatment, the samples were placed in an oven at 130 °C for 3, 6, 9, 12, 15, and 18 h, cooled naturally to room temperature 25 °C, making the cooling rate 3.5 °C/min and passed the 100 mesh sieve again. The starch samples were labeled CDH-3, CDH-6, CDH-9, CDH-12, CDH-15, and CDH-18. Among them, CDH-3 and RDH-1 are treated similarly, so RDH-1 is used to represent them uniformly.

### 2.3. Microscopy Analysis

#### 2.3.1. Scanning Electron Microscopy (SEM)

Starch samples were uniformly glued to a conductive adhesive covered with thin gold and carbon under vacuum conditions. The morphology and structure of starch samples were observed by a scanning electron microscope under a 5 kV acceleration voltage.

#### 2.3.2. Confocal Laser Scanning Microscopy (CLSM)

The starch samples (2 mg) were spread in a mixture of 4 μL 8-Aminopyrene-1,3,6-trisulfonic acid, trisodium salt (APTS) and 4 μL 1 M cyanoborohydride. The supernatant was removed after a constant temperature water bath at 30 °C for 15 h and centrifuged at 1500× *g* for 5 min. The residue was washed 5 times with 1 mL of distilled water, then suspended in a 20 μL solution of glycerol-water (1:1, *v/v*) and observed by CLSM.

### 2.4. Determination of Molecular Weight Distribution

The molecular weight distribution of starch samples was determined by High-Performance Liquid Chromatography. The starch sample (20 mg) and 0.1 mol/L NaNO_3_ (1 mL) solution were mixed evenly and heated in a boiling water bath for 5 min to dissolve completely. After cooling, the starch was filtered through a 0.22 μm aqueous phase filter. In addition, 20 μL of the liquid was purified through a 0.22 μm aqueous filter for determination by high-performance liquid chromatography.

According to Wang et al. [18], the molecular weight of starch samples was measured by High-Performance Liquid Chromatography (Waters, Model 410, Milford, MA, USA). The temperature of the column and detector was kept at 45 °C, the mobile phase was 0.1 mol/L NaNO_3_ solution and the flow rate was 0.60 mL/min. Dextran standard products with different molecular weights (purchased from Sigma, molecular weights of 5000, 12,000, 80,000, 15,000, 410,000, and 2,000,000 g/mol) were determined. The standard curve was:

Lg Mw = −0.0537 × (RT)3 + 2.0785 × (RT)2 − 27.186 × RT + 125.17 (R2 = 0.9992)(1)

### 2.5. X-ray Diffraction (XDR) Analysis

The diffraction pattern of starch crystals was measured by an X-ray diffractometer using CuKα as the radiation source. The scanning range of 2θ from 4° to 60°, the scanning speed of 6°/min and the step size of 0.02 were the operating conditions for X-ray diffraction analysis.

### 2.6. Solubility (S) and Swelling Power (SP) Measurement

The starch sample (0.6 g) was spiked into distilled water to prepare a solution with a mass fraction of 2%, and the solution was bathed at 50, 60, 70, 80, and 90 °C for 30 min, respectively. After cooling and centrifuging at 1500× *g* for 15 min, the supernatant was dried at 105 °C to a constant weight. The formula is as follows:

SP = weight of sediment × 100/weight of dry starch × (100 − solubility)(2)

S = weight of dried supernatant/weight of dry starch in the original sample × 100(3)

### 2.7. Light Transmittance Measurement

A starch suspension (1%) was made by adding 0.6 g of starch sample to distilled water and heating it in a boiling water bath until it was wholly pasted. Then, the starch paste was heated for 30 min and cooled to room temperature, and the transmittance was measured at 0, 24, 48, 72, 96 and 120 h using a UV-Vis spectrophotometer at 620 nm.

### 2.8. Pasting Properties Analysis

A Rapid Viscosity Analyzer was used to measure the pasting properties of starch. Then, according to the moisture content by the sample calculator, we calculated the amount of starch and water required. A specific mass of starch and 25 mL of distilled water were dispersed in an RVA aluminum canister. After shaking well, starch samples were put into the instrument and pressed on the column lid to start the measurement cycle program. The starch solution was balanced at 50 °C, held for 1 min, and the descending starch was heated to 95 °C to keep it at a rate of 12 °C/min. The starch samples were kept at 95 °C for 2.5 min before cooling to 50 °C and kept at 50 °C for 2 min at the same rate. The speed was 160 rpm during the measurement.

### 2.9. Differential Scanning Calorimetry (DSC)

The thermodynamic properties of starch were determined by differential scanning calorimetry. The starch samples weighed in the aluminum tray were mixed in 9 μL deionized water for sealing and equilibrating at 4 °C for 12 h. Then, the starch samples were placed in a DSC analysis that was heated from 20 °C to 120 °C at a rate of 10 °C/min.

### 2.10. In Vitro Digestibility Analysis

In vitro starch digestibility was determined according to Englyst et al. [19]. The method using 3,5-dinitrosalicylic acid (DNS) was used to determine the reducing sugars, RDS, SDS and RS of potato starch. The formula is as follows:

RDS% = (G_20_ − FG)/TS × 0.9 × 100 (4)

SDS% = (G_120_ − G_20_)/TS × 0.9 × 100 (5)

RS% = (T − RDS − SDS)/TS × 100(6)

where, TS is total starch weight; FG is the content of free glucose in starch; G_20_ and G_120_ are the contents of glucose released within 20 and 120 min of hydrolysis, respectively.

### 2.11. Statistical Analysis

All statistical analyses were performed using SPSS (20.0, IBM) software. Data were analyzed with analysis of variance (ANOVA), which allows for testing relationships between variables. All measurements were repeated 3 times, and differences between the mean values of samples were checked for significance (* *p* < 0.05). Origin Lab (Origin Pro 8.5) software was used to plot our results.

## 3. Results and Discussion

### 3.1. Morphological Structure

**Figure 1:**
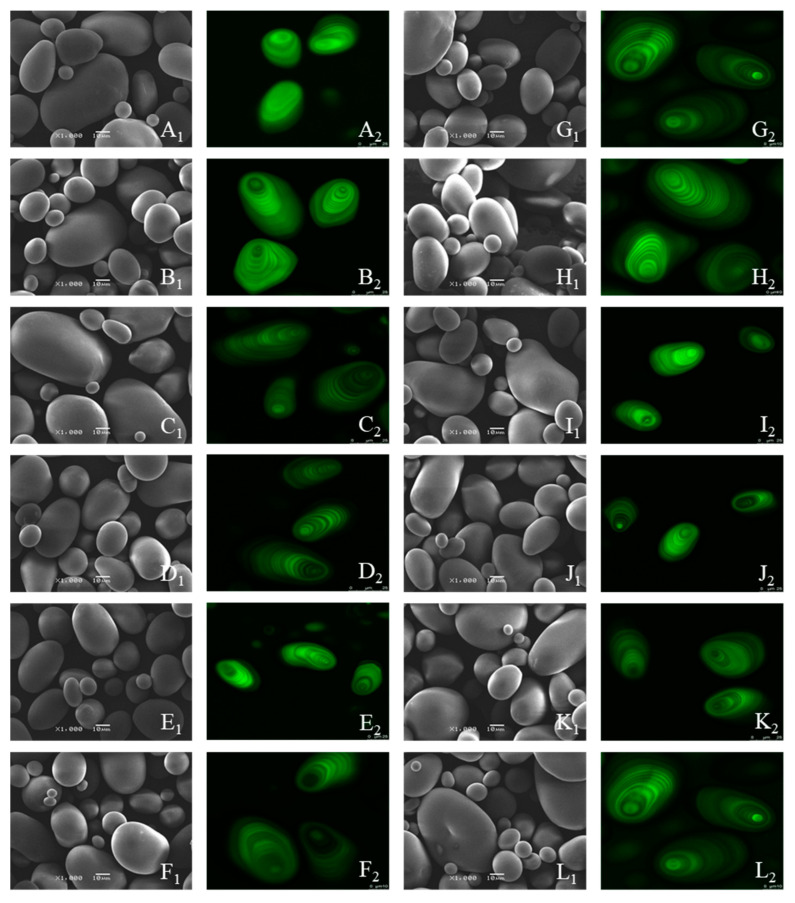
Scanning electron micrographs (SEM) (×1000) (1) and confocal laser scanning micrographs (CLSM) (512 × 512 pixel resolution) (2) of the native, CDH- and RDH-treated potato starch samples. (**A1**,**A2**) Native; (**B1**,**B2**) CDH-6; (**C1**,**C2**) CDH-9; (**D1**,**D2**) CDH-12; (**E1**,**E2**) CDH-15; (**F1**,**F2**) CDH-18; (**G1**,**G2**) RDH-1; (**H1**,**H2**) RDH-2; (**I1**,**I2**) RDH-3; (**J1**,**J2**) RDH-4; (**K1**,**K2**) RDH-5; (**L1**,**L2**) RDH-6.

SEM is a microscopic technique that characterizes the molecular structure of starch [20]. Figure 1 shows SEM images of the native and DHT potato starch samples. The native sample was oval, like a potato, with complete granules, a smooth surface, and different sizes, consistent with previous studies [21]. The starch samples maintained their original form, and only a few folds occurred on the surface of one end of the granules after CDH and RDH treatment, probably as explained by the leaching out of straight-chain starch after heating [12]. There is no noticeable variability with an increase in times and cycles. Agglomeration is another change in starch granule properties brought about by DHT. Some studies reported that the waxy corn starch granules aggregated into lumps and the surface was eroded after DHT [14], while the cassava starch granules agglomerated into clusters and cracked, which was due to the heating of amylose to make it leached [22]. The appearance of agglomeration is presumed to be related to water loss and changes in surface properties [10]. Furthermore, the difference between CDH and RDH starch samples at the same treatment time could not be clearly expressed.

CLMS images (Figure 1(A2)) display potato native starch with distinct growth rings composed of alternating amorphous and semi-crystalline shells [23]. The hilum of starch is the beginning of the starch granule growth. The amylose content is high if the umbilical point’s fluorescence intensity is strong. However, starch samples showed relatively weak APTS fluorescence properties, attributed to the effect of potato starch’s relatively low amylose content [24]. The growth ring structure of potato starch granules and clarity and brightness did not change significantly after DHT. Consistent with the SEM results, the differences between CDH and RDH starch samples could not be substantially expressed in the same treatment time (Figure 1(B2–L2)).

### 3.2. Molecular Weight (Mw) Analysis

According to Table 1, the molecular weights (Mw) of potato native starch and its RDH and CDH-treated starches can be obtained. The Mw of potato native starch and its processed starches are composed of two elution peaks, mainly amylopectin and amylose. Peak Ι indicates the amylopectin that was eluted first due to having a larger Mw, and then amylose is marked as Peak II [25]. As shown in Table 1, as the cycle number of RDH increased, the Mw of Peak Ι of potato starch decreased to a minimum of 5.61 × 10^16^ g/mol in the first four cycles. Similarly, the Mw of Peak II decreased continuously (RDH-1-RDH-4, CDH-3-CDH-12), attaining a minimum value at RDH-4 and CDH-12, respectively, and then inclined, but still smaller than that of native starch. The decline in the molecular weight of potato starch may be related to the cleavage of straight and straight chains in starch by DHT, which shortened the molecular chain and led to a smaller molecular weight. The subsequent increase in Mw may be caused by the rearrangement of amylose and amylopectin, increasing the degree of starch ordering.

Overall, the Mw of peak I was significantly greater for starch with RDH than for CDH-treated, a result that followed the same trend as for mung bean starch with CDH, probably owing to the more significant disruption of amylopectin by RDH, which reorganized the damaged starch [11]. In addition, this result suggests that starch treated with CDH is more likely to reach the equilibrium point [26].

### 3.3. X-ray Diffraction Patterns

**Figure 2:**
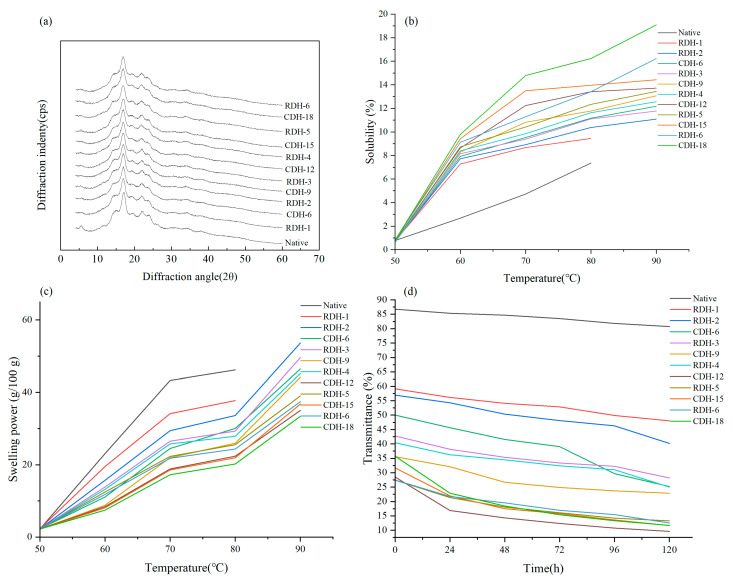
X-ray diffraction patterns (**a**), Solubility (**b**), swelling power (**c**) and transmittance (**d**) of the native, CDH and RDH-treated potato starch samples.

The X-ray diffraction pattern indicates an ordered phase mainly determined by the arrangement of amylose [27]. Potato native starch showed sharp diffraction peaks around 5°, 17°, 20°, 22°, and 24°, and the diffraction peaks that appeared in other regions were diffuse, which indicated that the native starch had a B-type crystalline structure. (Figure 2a) [28]. After CDH treatment, the starch crystalline form remained unchanged since there was no change in the position of the diffraction peaks. Most studies have shown that the crystalline type of grain starch generally does not alter after DHT, but the intensity of the diffraction peak changes. Quinoa starch [16] and chestnut starch [29] also maintained their crystal morphology after DHT, probably stemming from the fact that the alteration of starch properties was mainly in the amorphous region of the granules [15].

Compared with native starch, RDH and CDH treatments increased the crystallinity of starch in an overall trend, leading to a better microcrystalline structure and a more orderly rearrangement of starch molecules [30]. Liang et al. [11] reported that crystallinity increased due to the destruction of part of long amylopectin and the increase of short amylose content by DHT. At the same time, the crystalline region of starch granules was mainly composed of amylose. With repeated cycle times, the crystallinity of potato starch increased to a maximum of 46.74% at RDH-4 and then decreased to the minimum (36.35%). In contrast, the crystallinity dropped to the lowest value of 19.68% at CDH-9 and then rose from CDH-12 to CDH-18. Disruption of the crystalline region of starch, or partial reorganization of starch in the amorphous region by DHT may lead to a reduction in crystallinity [8]. Other than that, the relative crystal structure of RDH-treated starch was higher than that of CDH-treated (Table 1), contrary to the results reported for sweet potato [15], millet [8] and waxy corn starches [14]. Compared to the maize starch treated with RDH, the CDH-treated starch was significantly higher. This may be due to repeated heating and cooling during RDH, which restricted the starch molecular chain to forming an unstable crystal structure [12].

### 3.4. Starch Solubility and Swelling Power

The solubility and swelling power (SP) of potato starch and DHT starch at different temperatures can be seen in Figure 2b,c. Starch granules, straight-chain starch content, molecular weight, and crystallinity affect solubility and SP [16]. The solubility of both treatment and native starch improved gradually as the temperature increased from 50 to 90 °C. Similarly, the solubility of sweet potato starch [15] and mung bean [11] starch increases when the temperature increases (50 °C to 90 °C). On other side, the solubility of starch improved as the treatment time and cycles increased under the same temperature, reaching maximum values for both RDH-6 and CDH-18 at 90 °C. It may be attributed to the damage to the amorphous region and the surface of starch granules during DHT, which dissociated and diffused out amylose, characterizing the transition from ordered to disordered within the starch granules, resulting in increased starch solubility [15]. In addition, possibly due to the reduction of starch molecular interactions and starch diffusion by RDH treatment, the starch is lower in solubility compared with CDH, a finding consistent with mung bean starch [11]. The results showed that the effect of CHD treatment on potato starch structure was more significant than RDH.

Potato native starch has an excellent expansion force. The main reason is that the granule and crystalline area have ample space, and the phosphate group charges are constantly repelling each other, making the starch granules expand continuously. Therefore, under similar processing, the SP of native starch and DHT-treated starch went up with the increase in temperature. At 50 °C, the starch samples’ SP was small and did not begin to gelatinize; therefore, there is not much difference between native and DHT starch. Still, at 60–90 °C, the starch was gelatinized entirely, and the SP increased. Furthermore, the SP of CDH- and RDH-treated starches reduced with increasing treatment time and cycles at the same temperature (except 50 °C). This behavior is ascribed to the realignment and recombination of starch chains, reducing hydrotropic capacity and limiting swelling forces [31]. Meanwhile, the lower SP of starch after CDH treatment may result from the high crystallinity and dense crystal structure of potato starch, resulting in its low SP [11].

### 3.5. Light Transmittance

Light transmittance indicates the transparency of starch paste, reflecting the retrogradation process of starch [32]. It can be seen from Figure 2d that the values of percentage light transmittance of all starch samples gradually decreased with the standing time from 0 to 120 h, which was consistent with the results of sweet potato starch [15] under the same treatment conditions. This phenomenon was attributed to the progressive retrogradation of starch during storage.

The light transmittance of starch treated with RDH or CDH decreased simultaneously and increased slightly after reaching the minimum value when treated with CDH-12 and RDH-5. The decrease in light transmittance may be due to the leaching of amylose by the breakdown of starch granules under heating [33]. Conversely, the increase in transparency is probably owing to the decrease in the interaction between starch molecules as the treatment time and cycles increase, making the molecules disperse without swelling [9]. In addition, the transmittance of potato RDH-treated starch was higher than that of CDH-treated starch under the same treatment time. Similarly, a finding was reported by Zou et al. [14], who observed that waxy corn starch showed the same results under the RDH and CDH treatment conditions. The result revealed that RDH-treated starch damaged the starch more strongly during heating, making amylose diffuse.

### 3.6. Pasting Properties

The pasting features of potato native starch and DHT starch were measured by RVA. PV stands for peak viscosity, TV for trough viscosity, FV for final viscosity, BD for breakdown, SB for the setback, PT for peak time, and GT for pasting temperature.

For the changes in PV, FV and BD, DHT altered potato starch consistently and less than native starch, and these values dropped off with increasing treatment time and cycles (Table 2). When Liu et al. [29], Zou et al. [14], and Gou et al. [15]. studied the DHT of chestnut, waxy corn and sweet potato starch, PV, TV, and FV also showed a decreasing trend. The PV and FV represent the maximum viscosity and the cold paste viscosity, respectively [34]. PV is related to the swelling force and water-binding ability of starch [35]. Enhanced binding between potato starch chains and intracranial binding during DHT leads to a decrease in PV [15], suggesting that the structure of the starch is disrupted [14], which is consistent with the swelling force results. On the contrary, FV represents the cold paste’s stability, which reflects the ability of starch molecules to form a paste in the process of cooling [36], and the leaching of amylose will also affect FV [26]. In addition, TV represents the lowest viscosity, which correlates with the breakdown of starch granules [37]. Both FV and TV of DHT starch dropped with treatment and the number of cycles, which might be caused by the expansion and rupture of starch granules at the end of heating [37]. Meanwhile, the BD and SB values of starch during this period showed opposite trends before RDH-4 and CDH-12; the BD value of RDH treatment was greater than that of CDH, while the SB value was opposite, which the trend of SB was the same as that of quinoa starch in DHT [16]. SB expresses the difference between FV and TV, representing the cold starch paste’s degree of aging and stability. The lower the setback, the less likely the starch will deteriorate [15]. At the same time, PT and GT showed rising stages during the dry heat treatment. These results indicated that DHT could enhance the thermal stability of potato starch.

The PV, TV, FV, BD, and SB of RDH-treated starch were more than those of CDH for the same treatment time. The study reported that PT, TV and TV of sweet potato starch were the same as potato starch under RDH and CDH treatment. This indicates that CDH treatment increases the intramolecular bonding force more strongly than RDH, making starch paste less viscous [15]. Compared with CDH, RDH also showed higher BD and SB values, and waxy corn starch showed the same trend under the same treatment, implying that RDH treatment had weaker shear resistance [14]. Finally, there was little variability between the peak time of starch and the pasting temperature.

### 3.7. DSC Analysis

**Figure 3:**
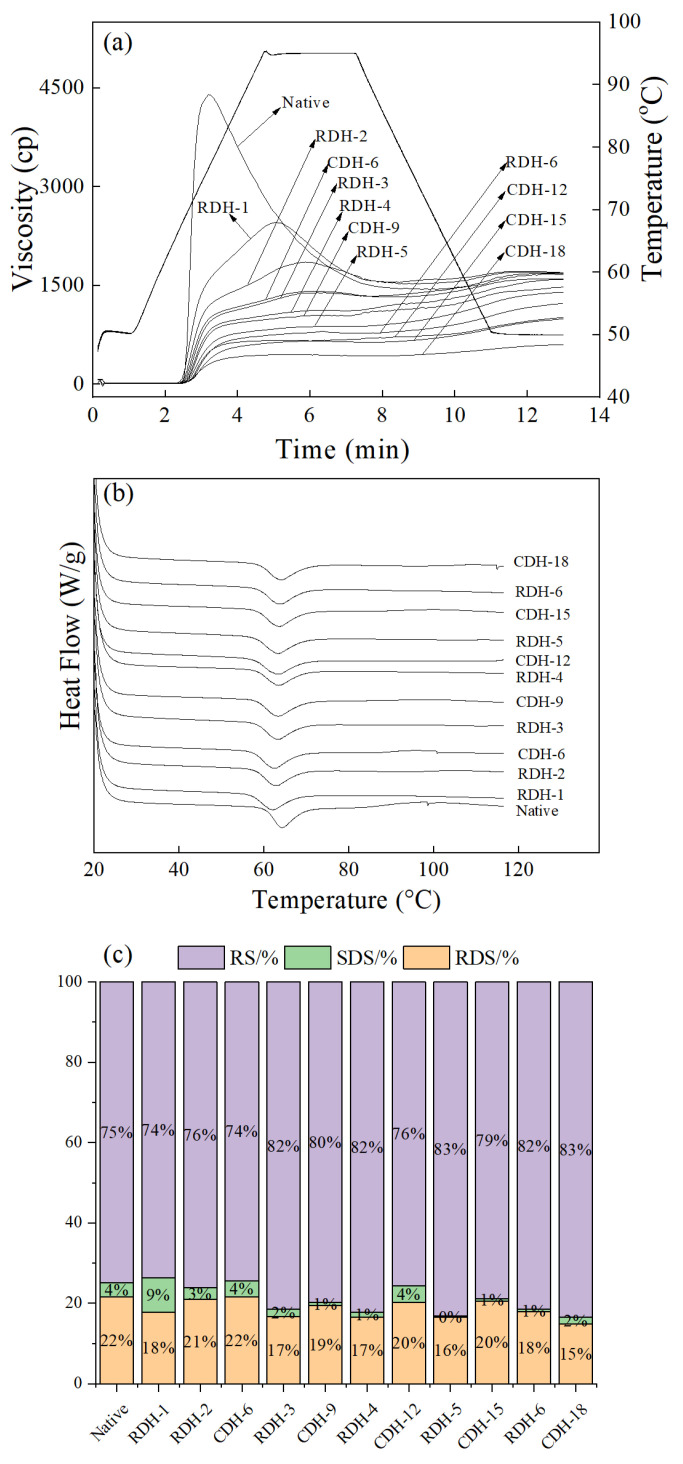
Pasting properties (**a**), DSC thermograms (**b**) and RDS, SDS and RS (**c**) of the native, CDH- and RDH-treated potato starch samples.

DSC is a thermal analysis method that refers to the thermal energy transformations that happen when the hydrogen bonds in the gelatinized starch molecules are broken under the heating of excess water [38]. The thermal properties of potato native starch and DHT starch are presented in Table 3 and Figure 3b. In general, the endothermic peak of potato starch showed a shift to the left after the DHT, reducing the thermodynamic temperature of the starch (Figure 3b). Similarly, the thermodynamic temperature of waxy corn starch declined after DHT, probably due to the destruction of amylose [14].

As seen in Table 3, the potato starch showed a slightly lower initial temperature (T_o_) and peak temperature (T_p_) than native starch after DHT. Similar results were also reported for high-amylose rice starch [13], tapioca starch [22] and glutinous rice starch [39]. T_o_, T_p_ and T_c_ demonstrate the integrity of the starch double helix sequence [40], where T_o_ is the temperature at which the lowest crystalline structure melts. T_c_ is the highest-temperature crystalline structure that melts [41]. The reduction of T_o_, T_p_, T_c_, and ΔH may be because the molecular chain of starch was broken, and the amorphous region was destroyed during DHT, while moisture reached the interior of starch more conveniently [42]. As treatment time and cycling increased, the thermodynamic parameters (T_o_, T_p_, T_c_) of potato starch showed an increasing trend. The increase of T_o_, T_p_ and T_c_ values indicated that DHT could strengthen the order of the crystal structure and the thermal stability of starch was, to some extent, improved. Gou et al. [15] had similar results for heat-treated sweet potato. At the same time, the increase of double helix sequence content made ΔH larger, which the rearrangement of destroyed molecules may cause during processing [43,44]. Simultaneously, under the same treatment time, there was no significant difference in the thermodynamic parameter values of potato starch between RDH and CDH treatment starch.

### 3.8. In Vitro Digestibility

Table 4 and Figure 3c list the amounts of RDS, SDS, and RS of native potato starch and DHT starch. Overall, the RDH and CDH treatments resulted in lower levels of RDS than the native starch, a trend identical to that previously found in waxy potato starch [15]. However, in contrast to the results in other studies for quinoa starch [16] and waxy potato [14], the RDS content of starch compared to native starch increased after DHT. Meanwhile, RDS reached the minimum value at RDH-5 (16.49%) and CDH-18 (14.81%), respectively. The reduced content indicated that the DHT lowered the overall digestibility of potato starch, and the same trend was also observed for chestnut starch [29] and high amylose rice starch [13] after DHT.

Furthermore, the content of RS was slightly higher than potato native starch, among which RS exhibited an increasing trend before CDH-9 (79.73%) and RDH-5 (83.21%). The higher RS is due to the enhanced interactivity between the microcrystalline structure of starch and starch chains, inhibiting the entry of enzymes into the interior to hydrolyze it. In addition, the SDS content increased slightly after decreasing to a minimum of 0.3% and 0.64% with the number of treatment times and cycling (except CDH-12). The reorganization of starch molecular chains to improve the crystal structure may lead to a decrease in SDS content [37], while the increased sensitivity of starch explains the slight increase in SDS to enzymatic digestion [42]. It should be noted that during that time, the RDS content of samples treated with RDH was generally lower than that of starch treated with CDH. It can be found that RDS and RS always show opposite trends [17]; hence, the increase in RDS may be due to the RS conversion obtained in part. It is also demonstrated that RDH is more favorable to reduce the digestibility characteristics, which may be assigned to the reorganization of starch microcrystals as the starch is cooled.

## 4. Conclusions

The significant effects of RDH and CDH treatments on potato starch structure and physicochemical and digestive properties were investigated. The structural properties of starch, such as morphology and crystalline shape, remain the same. The crystal form was still the original B type during the processing. However, DHT changed other properties of the starch. It not only reduced both swelling power and light transmittance of the starch lower than the native starch, but also increased solubility and descended with temperature. PV, FV, BD and thermal properties (To, Tp, Tc, ΔT) decreased during the treatment, indicating that the DHT could improve the thermal stability of the starch. Besides, RDH-treated starch exhibited higher transparency, RS, and peak viscosity than CDH-treated starch under the same treatment time. In a word, RDH is a dry heat treatment performed again after starch reaches equilibrium with the external environmental conditions, which significantly impacts the starch. These results are expected to contribute to the widespread use of dry heat-modified starches in food processing.

## Figures and Tables

**Table 1:** Molecular weight and XRD patterns of the native, CDH- and RDH-treated potato starch samples.

Sample	Molecular Weight g·mol^−1^		RC (%)	Crystal Type
	Peak Ι	Peak II		
Native	1.75 × 10^20^	3.26 × 10^12^	27.27 ± 0.30 ^g^	B
RDH-1	2.93 × 10^19^	3.57 × 10^12^	36.42 ± 0.30 ^e^	B
RDH-2	1.89 × 10^19^	3.57 × 10^12^	43.25 ± 0.17 ^c^	B
CDH-6	1.23 × 10^19^	2.07 × 10^12^	32.40 ± 0.18 ^f^	B
RDH-3	6.65 × 10^17^	1.29 × 10^11^	44.60 ± 0.33 ^b^	B
CDH-9	3.95 × 10^18^	1.02 × 10^12^	19.68 ± 0.25 ^i^	B
RDH-4	5.61 × 10^16^	7.47 × 10^10^	46.74 ± 0.21 ^a^	B
CDH-12	1.78 × 10^17^	5.51 × 10^10^	25.01 ± 0.44 ^h^	B
RDH-5	6.03 × 10^18^	1.29 × 10^11^	37.82 ± 0.06 ^d^	B
CDH-15	3.00 × 10^16^	6.41 × 10^10^	32.50 ± 0.43 ^f^	B
RDH-6	1.50 × 10^18^	6.09 × 10^11^	36.35 ± 0.11 ^e^	B
CDH-18	1.20 × 10^17^	5.14 × 10^11^	36.77 ± 0.13 ^e^	B

Values are means ± SD. Values with the same letters within the same column are not significantly different (*p* < 0.05).

**Table 2:** Pasting parameters of the native, CDH- and RDH-treated potato starch samples.

Sample	PV/cp	TV/cp	FV/cp	BD/cp	SB/cp	PT/min	GT/°C
Native	4437 ± 53 ^a^	1444 ± 9 ^b^	1667 ± 6 ^a^	2994 ± 45 ^a^	223 ± 3 ^e^	3.20 ± 0.00 ^f^	67.80 ± 0.07 ^cde^
RDH-1	2451 ± 1 ^b^	1517 ± 35 ^a^	1683 ± 14 ^a^	935 ± 33 ^b^	167 ± 21 ^f^	5.10 ± 0.04 ^e^	66.50 ± 0.64 ^f^
RDH-2	1843 ± 13 ^c^	1470 ± 68 ^ab^	1665 ± 25 ^a^	373 ± 55 ^c^	195 ± 43 ^ef^	5.70 ± 0.24 ^de^	66.97 ± 0.04 ^ef^
CDH-6	1420 ± 19 ^d^	1327 ± 9 ^c^	1597 ± 9 ^b^	93 ± 10 ^d^	271 ± 1 ^d^	6.00 ± 0.18 ^bcd^	67.42 ± 0.67 ^def^
RDH-3	1354 ± 44 ^e^	1297 ± 50 ^c^	1655 ± 30 ^a^	58 ± 6 ^de^	358 ± 20 ^c^	6.07 ± 0.19 ^bcd^	67.80 ± 0.07 ^cde^
CDH-9	1028 ± 23 ^g^	1009 ± 38 ^e^	1463 ± 9 ^c^	19 ± 16 ^e^	454 ± 29 ^b^	5.77 ± 0.71 ^cde^	68.22 ± 0.60 ^cd^
RDH-4	1112 ± 12 ^f^	1097 ± 14 ^d^	1566 ± 32 ^b^	15 ± 2 ^e^	465 ± 18 ^b^	6.53 ± 0.28 ^abc^	67.80 ± 0.07 ^cde^
CDH-12	668 ± 8 ^j^	656 ± 0 ^h^	1012 ± 10 ^f^	12 ± 8 ^e^	356 ± 10 ^c^	6.67 ± 0.19 ^ab^	70.17 ± 0.04 ^a^
RDH-5	872 ± 7 ^h^	865 ± 6 ^f^	1387 ± 5 ^d^	8 ± 1 ^e^	522 ± 1 ^a^	6.97 ± 0.05 ^a^	68.60 ± 0.07 ^bc^
CDH-15	661 ± 2 ^j^	631 ± 2 ^h^	979 ± 9 ^f^	30 ± 0 ^e^	349 ± 6 ^c^	5.04 ± 0.05 ^e^	69.37 ± 0.11 ^ab^
RDH-6	778 ± 32 ^i^	758 ± 16 ^g^	1211 ± 13 ^e^	20 ± 16 ^e^	454 ± 4 ^b^	6.07 ± 0.37 ^bcd^	69.40 ± 1.06 ^ab^
CDH-18	458 ± 16 ^k^	437 ± 18 ^i^	628 ± 45 ^g^	22 ± 2 ^e^	192 ± 28 ^ef^	5.97 ± 0.62 ^bcd^	70.25 ± 0.00 ^a^

Values are means ± SD. Values with the same letters within the same column are not significantly different (*p* < 0.05).

**Table 3:** Thermal properties of the native, CDH- and RDH-treated potato starch samples.

Sample	T_o_/°C	T_p_/°C	T_c_/°C	ΔT/°C	ΔH/J·g^−1^
Native	60.83 ± 0.31 ^a^	64.05 ± 0.25 ^a^	72.21 ± 0.18 ^ab^	11.38 ± 0.49 ^d^	14.10 ± 0.15 ^a^
RDH-1	57.18 ± 0.08 ^e^	61.80 ± 0.23 ^g^	69.72 ± 0.69 ^d^	12.54 ± 0.61 ^bc^	12.33 ± 0.24 ^f^
RDH-2	58.20 ± 0.23 ^cd^	62.71 ± 0.27 ^f^	70.66 ± 0.50 ^cd^	12.46 ± 0.27 ^c^	12.72 ± 0.10 ^ef^
CDH-6	57.79 ± 0.13 ^d^	62.54 ± 0.20 ^f^	71.28 ± 0.25 ^bc^	13.49 ± 0.37 ^ab^	13.65 ± 0.40 ^abc^
RDH-3	58.63 ± 0.05 ^b^	63.13 ± 0.10 ^de^	71.77 ± 0.81 ^ab^	13.14 ± 0.86 ^abc^	13.33 ± 0.18 ^bcde^
CDH-9	58.50 ± 0.15 ^bc^	62.87 ± 0.30 ^ef^	71.81 ± 0.75 ^ab^	13.32 ± 0.60 ^abc^	13.43 ± 0.35 ^abcd^
RDH-4	58.70 ± 0.11 ^b^	63.36 ± 0.04 ^cd^	72.39 ± 0.19 ^a^	13.69 ± 0.30 ^a^	12.86 ± 0.42 ^def^
CDH-12	58.12 ± 0.13 ^cd^	63.29 ± 0.06 ^d^	72.12 ± 0.44 ^ab^	14.00 ± 0.57 ^a^	13.13 ± 0.52 ^cde^
RDH-5	58.47 ± 0.28 ^bc^	63.39 ± 0.22 ^cd^	72.35 ± 0.37 ^a^	13.88 ± 0.10 ^a^	13.24 ± 0.19 ^bcde^
CDH-15	58.74 ± 0.01 ^b^	63.54 ± 0.18 ^bcd^	72.26 ± 0.25 ^ab^	13.52 ± 0.26 ^ab^	13.85 ± 0.10 ^ab^
RDH-6	58.83 ± 0.15 ^b^	63.72 ± 0.05 ^abc^	72.43 ± 0.25 ^a^	13.61 ± 0.40 ^a^	13.28 ± 0.35 ^bcde^
CDH-18	58.84 ± 0.33 ^b^	63.85 ± 0.04 ^ab^	72.56 ± 0.44 ^a^	13.72 ± 0.11 ^a^	13.87 ± 0.40 ^ab^

Values are means ± SD. Values with the same letters within the same column are not significantly different (*p* < 0.05).

**Table 4:** RDS, SDS and RS of the native, RDH- and CDH-treated potato starch samples.

Sample	RDS/%	SDS/%	RS/%
Native	21.55 ± 0.17 ^a^	3.57 ± 0.08 ^c^	74.88 ± 0.47 ^g^
RDH-1	17.73 ± 0.14 ^f^	8.60 ± 0.10 ^a^	73.67 ± 0.22 ^i^
RDH-2	20.96 ± 0.08 ^b^	3.00 ± 0.05 ^d^	76.04 ± 0.06 ^e^
CDH-6	21.67 ± 0.08 ^a^	3.88 ± 0.06 ^b^	74.45 ± 0.12 ^h^
RDH-3	16.74 ± 0.10 ^g^	1.74 ± 0.03 ^e^	81.52 ± 0.11 ^c^
CDH-9	19.42 ± 0.06 ^d^	0.85 ± 0.06 ^g^	79.73 ± 0.08 ^d^
RDH-4	16.55 ± 0.06 ^h^	1.18 ± 0.15 ^f^	82.27 ± 0.11 ^b^
CDH-12	20.28 ± 0.06 ^c^	4.02 ± 0.19 ^b^	75.70 ± 0.17 ^f^
RDH-5	16.49 ± 0.03 ^h^	0.30 ± 0.00 ^i^	83.21 ± 0.03 ^a^
CDH-15	18.44 ± 0.08 ^e^	0.64 ± 0.00 ^h^	71.12 ± 0.17 ^j^
RDH-6	17.82 ± 0.03 ^f^	0.63 ± 0.00 ^h^	81.47 ± 0.15 ^c^
CDH-18	14.81 ± 0.06 ^i^	1.76 ± 0.12 ^e^	83.44 ± 0.08 ^a^

Values are means ± SD. Values with the same letters within the same column are not significantly different (*p* < 0.05).

## Data Availability

The data presented in this study are available on request form the corresponding author.
